# Editorial: Research challenges of drug utilization, data collection, data validation, and adverse drug reactions in neonates

**DOI:** 10.3389/fphar.2024.1376770

**Published:** 2024-03-12

**Authors:** Robert M. Ward

**Affiliations:** Department of Pediatrics, University of Utah, Salt Lake City, UT, United States

**Keywords:** neonatal pharmacology, adverse events newborns, parabens in newborns, clinical pharmacists, postnatal corticosteroids in newborns, probiotics for antibiotic associated diarrhea, medication error screening by computer, ocular pressure newborns post injection

Federal laws in the U.S. since 1997 have both incentivized and required pediatric studies of new medications, leading to over 1,000 pediatric label changes. AAP News (2022) However, newborns have not seen any significant benefit from these laws. Of the few drugs studied through these laws, Laughon et al. pointed out that two-thirds of the drugs studied are never or rarely used in the neonatal intensive care unit (NICU). Laughon et al. (2014) The non-profit International Neonatal Consortium (INC) has developed a series of white papers outlining the need for the study of drugs in newborns and descriptions of how to study various neonatal disorders (International Neonatal Consortium, 2015). This issue of Frontiers in Pharmacology includes some articles covering work by the INC and others about how to use and study drugs in newborns while outlining important issues needing study.

Neonatal drug studies are uniquely challenging due to their immaturity, small size, developmental changes in physiology and pharmacology, unique diseases, and parental stress, leaving clinicians to use most drugs off-label in the NICU. Ward et al. (2017) To select drugs and determine dosages, clinicians often depend on recommendations from experts and published studies compiled in handbooks. Drug therapy for newborns should incorporate reports of adverse drug reactions (ADRs). As used here and throughout the editorial, adverse drug reactions are those that are known to be caused by a medication. In drug regulations, adverse drug events are changes coinciding with drug treatment and may or may not be caused by that drug. Detection of ADRs in newborns can be hard to separate from their illnesses. Yalcin et al. developed and validated a machine learning detection system to detect medication errors based on 10 clinical conditions, from workload to the number of medications prescribed. This is the first report of a machine learning algorithm to detect medication errors in the NICU, which should improve neonatal drug therapy.

Corticosteroid treatment of newborns has been shown to have a challenging history, beginning with a 6-week course of dexamethasone, which improved chronic lung disease and successful extubations but caused severe neurologic injury and developmental delay. Although it was not clear whether it was the dose or the drug, the American Academy of Pediatrics recommended that dexamethasone be avoided in newborns. Lower doses for shorter courses were later shown to be safe and effective, as was hydrocortisone. Concern about corticosteroid treatment of newborns persists, and many clinicians recommend against their use until the newborns are older. Iacobelli et al. analyzed postnatal corticosteroid exposure for 5 years in France at 41/68 tertiary NICUs, including almost 14,000 premature newborns <32 wk. Their computerized order entry system recorded the drug, dosage, and route. Postnatal corticosteroid treatment ranged from 5% to 56% of patients. Hydrocortisone was prescribed most often and increased during the study, usually to prevent or treat bronchopulmonary pulmonary dysplasia.

Pediatric clinical trials for FDA approval and labeling require reporting of adverse events (AEs). Until recently, the only severity grading system in use was designed for adult activities. Salaets et al. published the Neonatal Adverse Event Scoring System (NAESS) in 2019 with 35 categories of neonatal adverse events (AEs) with severity ranging from 1 to 5. Salaets et al. (2019) The current study evaluated the effectiveness of NAESS by having two individuals score 120 medication-related and medication-not-related events independently, both before and after a training session. NAESS improved interrater reliability.

Newborns have a history of adverse reactions to excipients in medications. Parabens have been identified as endocrine disruptors in animal models. In a 5-year study at two tertiary NICUs, Iacobelli et al. found neonatal exposure to parabens exceeded the European Medicines Agency Acceptable Daily Intake in 3.5% of patients. Five drugs were the leading cause of the exposures. Long-term effects are still unclear, but this exposure is worrisome and requires further evaluation.

Infections are common illnesses in newborns and infants. Antibiotic treatment is often associated with diarrhea (AAD), sometimes causing premature discontinuation of treatment. Despite limited supportive data, probiotics are frequently used to treat AAD. Yang et al. extracted 20 systematic reviews (SRs) of AAD treated with probiotics and analyzed the quality of the data. Their meta-analysis showed that 3 SRs had moderate quality data supporting high-dose treatment with *Lacticaseibacillus rhamnosus* and *Saccharomyces boulardii* to prevent AAD. They pointed out that other probiotics may also be effective.

Retinopathy of prematurity is an important, potentially sight-limiting problem in the extremely premature population of newborns who now survive. Vascular proliferation is often treated with intravitreal injections of drugs, such as conbercept, to inhibit vascular endothelial growth factor. Gao et al. studied the safety of these intravitreal injections by measuring the intraocular pressure. Although the pressure increased immediately after intravitreal injection of conbercept, it decreased within 2 h and remained low after that.

The limited study and labeling of medications for newborns, especially extremely premature newborns, make it hard for clinicians in the NICU to remain knowledgeable about correct dosages, adverse drug reactions, and drug–drug interactions. Off-label prescribing remains the rule rather than the exception in the NICU. Using a unique, blinded, randomized study design, Yalcin et al. demonstrated that clinical pharmacists’ input reduced medication errors, adverse drug reactions, and clinical drug–drug interactions. The special expertise of clinical pharmacists can improve the quality and safety of care in the NICU.

The detection and treatment of pain and agitation in neonates remain complicated. Rao and Oschman provided a comprehensive review of the management of pain, agitation, and opioid hyperalgesia in infants. This complex area of drug therapy in the NICU is challenging and unfortunately needs much more study.
